# Does tension-type headache patients have a reduced shoulder muscle strength compared to healthy controls?

**DOI:** 10.1186/1129-2377-14-S1-P52

**Published:** 2013-02-21

**Authors:** KB Madsen, LL Andersen, J Skotte, R Jensen

**Affiliations:** 1 Danish Headache center Department of Neurology Glostrup Hospital., Denmark; 2 Danish National research centre for the working environment, Denmark

## Introduction

Tension-type headache (TTH) is the most prevalent headache in the general population. Neck muscles are tense and tender and pain in shoulder muscles is a pronounced complaint. Several studies have found that the trapezius play a major role in TTH and decreased strength capacity and lowered activity of the painful trapezius muscle by office workers with trapezius myalgia are reported.

## Objectives

To investigate if TTH patients have a reduced torque during shoulder abduction compared to age- and sexmatched matched healthy controls.

## Methods

60 TTH patients from a multidisciplinary headache center fulfilled the ICHD-2 criteria for frequent episodic or chronic TTH and 30 healthy matched controls were studied. The participants were lying supine on the floor on a thin mattress, with the dominant arm in 90 degree shoulder abduction and the wrist positioned on a force transducer. The participants were instructed to abduct the arm with maximal force as quickly as possible, 3 attempts were made with 60 sec in between tests. The data was registrated on a connected computer, and the torque value was calculated as force times moment arm.

## Results

60 TTH-patients (19 males, 41 females) with a mean age 34 years and TTH > 8 days/mth, 30 sex and age matched healthy controls completed. There was a numerical but not significant difference in torque between TTH patients (38,66 N × m) and healthy controls (44,32 N × m) during shoulder abduction (p=0.143).

## Discussion

We had hypothesized that TTH patients would have a significant reduced torque compared to healthy controls. The results indicate that the influence from the trapezius muscle in TTH headache patients will not result in a reduced torque in shoulder abduction. This also implies that TTH patients are not limited in the use of the shoulder due to pain in trapezius and other mechanisms must be investigated.

